# The Hospital. Nursing Section

**Published:** 1905-07-01

**Authors:** 


### The Hospital.

**Nursing Section.**

Contributions for this Section of "The Hospital" should be addressed to the Editor, "The Hospital" Nursing Section, 28 & 29 Southampton Street, Strand, London, W.C.

No. 979.—Vol. XXXVIII. SATURDAY, JULY 1, 1905.

### Notes on News from the Nursing World.

### NEW TRAINING SCHOOL IN INDIA.

The foundation-stone of a new training school for *dais* and nurses at Patiala was recently laid by Miss M. Smith, sister of Major Dunlop Smith, the political agent. The Council of Regency, having signified their wish to call the school after Lady Curzon in commemoration of her recovery and safe return to India, her Excellency has accepted the compliment.

### NURSING IN NEWFOUNDLAND.

It is two years ago since the training school for nurses was started at the General Hospital, St. John's, Newfoundland, and we are glad to hear that the standard of nursing has been steadily improving. The institution was at first a military hospital, and consisted of the older part of the building which now contains the doctors' house, the nursing superintendent's room, the nurses' and servants' quarters, and one ward for women. Some years later a wing was added for male patients, consisting of two public wards, a small private ward, bathrooms, and an operating-room now converted into a ward. In commemoration of Queen Victoria's Jubilee a new wing for women was built, including an operating theatre and laundry, the greater part of the cost being defrayed by the women of Newfoundland. The medical staff consists of a resident superintendent, two visiting surgeons, and two physicians. As it is the only hospital for the whole island there is no lack of patients. They come from all parts and at all hours of the day and night. It is an unusual occurrence to have an empty bed even for a few hours. Very often one patient is sitting down beside the bed waiting for the other to leave it. Couches are constantly brought into requisition, and when an urgent case comes in the question frequently is who can be turned out to lie on the couch or on the floor. The Convalescent Home for Women and the Hospital for Infectious Diseases, which is just finished and will be opened shortly, stand in the same grounds.

### QUEEN VICTORIA'S JUBILEE INSTITUTE FOR NURSES.

We learn, as we go to press, that Miss Amy Hughes has been appointed lady superintendent of the above institution.

### HOLIDAYS AT TOTTENHAM HOSPITAL.

During the period while Miss Margaret Fox has been matron of Tottenham Hospital she has introduced an innovation in the shape of an extra week's holiday to each of the sisters during the winter. Her idea was that a week after Christmas would give the sisters a much needed rest, and break the monotony of the winter for them. We are not surprised to learn that the additional holiday is much appreciated by the sisters, who are always recruited at Tottenham Hospital from the ranks of the nurses. Every one recognises the importance of a summer holiday for nurses, but a week in the winter also is, at present, quite the exception.

### GIFTS TO PATIENTS.

A rule of considerable importance has been rescinded by the managers of Poplar and Stepney Sick Asylum. Hitherto, the friends of the patients have been prohibited from bringing them articles of food, but in future, at the suggestion of the medical superintendent, this rule will not be enforced. In order, however, to prevent abuses—or in the hope of doing so—any little delicacy brought by the friends of a patient must be handed to the sister, or nurse, in charge of the ward, and it will be for the latter to accept or reject the gift, as she or the medical officer thinks fit. We are afraid that this concession to the friends of the patients will materially add to the responsibilities of the sister or nurse, and may also give rise to friction. Of course, intoxicating liquors will not be accepted, but even so, a considerable number of gifts which would be injurious to the patients are sure to be proffered, and their rejection is equally certain to give rise to complaints.

### PUBLIC EXAMINATION FOR PROBATIONERS.

At a meeting of the Portsmouth Guardians, which was held last week, the report of the annual examination of nurses at the Infirmary was adopted. But it gave rise to a discussion of considerable length, in the course of which one of the guardians said he thought that the time had come when a syllabus and list of qualifications might be prepared and the appointments of probationers be competed for by public examination. With regard to a syllabus he was informed that a sub-committee had been appointed to draw up one; but the other part of the suggestion did not receive any encouragement. The fact is that there is no necessity for competition by public examination. If, as at Portsmouth Infirmary, there is a fully trained matron at the head of the school, she is perfectly competent to adjudicate upon the applications of young women anxious to serve as probationers. This is the system in force at all the great hospitals, and there is no reason why it should not answer just as well in Poor-law infirmaries which are run on up to date lines.

### THE SECTARIAN SPIRIT IN DISTRICT NURSING.

In connection with our recent remarks under the above heading, the *Christian World*, while endorsing our expression of regret that sectarian issues should be associated, as they unfortunately are at Chester, with the nursing of the sick, goes on to say, "In many a town and village throughout the land the representatives of the Established Church for long attempted entirely to annex the nurses for the poor provided by public subscriptions," and adds, "these nurses have been, and are, used as direct agents of the Church." We repeat that it is a matter for regret that the Church people and Nonconformists of Chester cannot set aside religious differences in the pursuit of such an object as the nursing of the sick poor. But, with some knowledge of facts, we strongly demur to the statement of the *Christian World* implying that it is a common practice to use district nurses as direct agents of the Church. On the contrary, as our columns show week after week, the hundreds of district nursing organisations in the country are carried on, without hitch, by committees on which various religious bodies are represented; and, we believe, that among the nurses themselves there are, more or less, members of almost every denomination.

### THE VALUE OF GARDEN SALES.

WE direct the attention of managers of nursing organisations in need of funds, to the signal success which has again rewarded the promoters of the garden sale in aid of the Southport and Birkdale District Nursing Society and Invalid Food Kitchen. The result last year was highly satisfactory, the proceeds of the sale of work at the fête amounting to £120; but this year they reached £160, notwithstanding that rain fell in the afternoon, and a number of competitions had to be postponed. It is not, of course, every association which is fortunate enough to have such kind friends as Mr. and Mrs. Vernon, of Wyborne Gate, Birkdale, who possess not only beautiful grounds and have pleasure in lending them, but also extend their hospitality to visitors. When, however, it is possible to obtain such assistance, it should be sought. It is true that no bait to support an organisation for the nursing of the sick poor should be needed, but there are undoubtedly many persons who, while refusing to subscribe, will go and make purchases at a garden sale for the sake of enjoying an outing. Moreover, such gatherings bring the friends of the movement together, and tend to increase the interest in it even on the part of those who in any circumstances would render help.

### PRACTISING MIDWIFE AND MONTHLY NURSE.

IT having been pointed out to the secretary of the Central Midwives Board that considerable diversity of opinion appears to exist amongst local supervising authorities as to whether a certified midwife can be required to notify on Form 12, provided she acts merely in the capacity of a monthly nurse under the direction of a doctor, he has replied to the effect that "Any woman certified by the Central Midwives Board becomes, *ipso facto*, a midwife by the operation of Section XVIII. of the Midwives Act." He continues, "Being midwife, she is bound by all the rules of the Board if she practises as such. The obligation to give notice to the local supervising authority arises under the Act, not under the rules, and it only operates in the case of a woman who practises, or holds herself out as practising, as a midwife. If she does neither of these things, but merely practises as a monthly nurse under the direction of a qualified medical practitioner, she is not bound to notify the local supervising authority. If, however, on any occasion she acts otherwise than under medical direction, she is liable to the penalty prescribed by Section X. of the Midwives Act, for having omitted to give notice." The obviously safe course for a parish nurse who is a certified midwife is to register her name with the local supervising authority, even if she does not usually attend confinements without a doctor.

### ALLEGED NEGLECT OF DUTY.

THE Rotherham Guardians do not seem able to make up their minds whether they ought to call upon some of their charge nurses to resign, or whether the accusations against them of neglect of duty are groundless. A short time ago they passed a resolution exonerating the nurses from blame, but the charges having been investigated by a committee appointed at their instance, and the report being in favour of requiring the resignation of two of the nurses, they have again discussed the matter. Eventually they referred it back to the committee for further consideration. This sort of thing is not fair either to the superintendent nurse, who originally made the complaints, or to the nurses who are alleged to have misconducted themselves. The charges are either well founded or unfounded, and it is quite time that they should be finally dealt with.

### LIBEL ACTION BY A HOSPITAL MATRON.

THE LORD CHIEF JUSTICE OF IRELAND and a special jury were occupied for some time in hearing an action brought by Miss Janet F. Mayne, matron of the Limerick County Infirmary, against Mrs. Harriet O'Brien, one of the governors. The alleged libel was a charge made by the latter that Miss Mayne had thrown iodoform, or caused it to be thrown, over her clothes, and the questions put by the Lord Chief Justice to the jury were: "Did the plaintiff throw or procure the throwing of the iodoform?" and "Was Mrs. O'Brien guilty of malice?" The evidence was of a highly controversial and contradictory character, and in the result the jury found that Miss Mayne neither threw the iodoform nor was a party to the throwing of it; but they were unable to agree whether Mrs. O'Brien was guilty of malice in making statements reflecting on the matron. Miss Mayne's reputation has, at all events, been cleared by the proceedings, and, having read the evidence, we do not think that she need trouble herself any further in the matter. There is one part only which touches a question of general interest. Mrs. O'Brien got up a ball for the benefit of the Infirmary, and then complained of the nurses going to it "in *décolleté* dresses and dancing-pumps to take the floor with the best." Her view is that "they ought to go in their uniform." This certainly tends to show that Mrs. O'Brien is not in her element as the governor of a hospital.

### SCHOOL NURSING.

THE question of school nursing was raised at the annual meeting of the Gillingham Association. A proposal was brought forward that the Queen's nurses should attend the elementary schools in the town for minor ailments, and it was stated that the cost could be defrayed by assistance from the local education committee. The idea did not, however, meet with much favour. Several members said that in order to carry it into effect an extra nurse would be required, others that the ratepayers would object, and two at least treated it as a joke, one asking whether the feeding of the younger children was not included, and another suggesting that children should be put under glass cases. Notwithstanding this feeble pleasantry, it is well known that school nurses have been most useful in London and elsewhere. We can understand that the Gillingham Association shrink from action which might possibly involve them in extra financial obligations, but the proposal which they at present decline to entertain may on another occasion have a more sympathetic reception.

### QUEEN ALEXANDRA'S MILITARY NURSING SERVICE.

We are officially informed of the following changes in Queen Alexandra's Imperial Military Nursing Service:—Miss M. C. E. Newman, staff nurse, has been transferred to Netley. Sister M. M. Blakely has been transferred to Khartoum, Sister E. M. Pettle to Malta, Sister C. K. E. Steel to Harrismith, Sister S. L. Wilshaw (R.R.C.) to Khartoum, and Sister M. Worthington to Cairo. Miss L. Belcher, Miss C. T. Bilton, Miss E. C. Fox, Miss M. O'C. McCreery, Miss M. E. Neville, and Miss A. Willes, staff nurses, have been confirmed in their appointments.

### THE MATRONSHIP OF SALISBURY INFIRMARY.

At a special Court of Governors of the Salisbury Infirmary, a resolution was unanimously passed recognising the services of Miss McMaster as matron during the past six and a half years. Canon Sir James Philipps, in seconding the motion, said that Miss McMaster had thrown herself heart and soul into the duties of her office, and had been a most capable matron. With regard to her successor, Lord Radnor, the president, stated that there were 60 applications for the vacant appointment, and it was subsequently decided to leave the choice in the hands of the Committee of Management with the addition of two outside governors. The election took place on Saturday and the successful candidate was Miss Vezey, at present assistant matron of St. Thomas's Hospital. Miss Vezey was for four years matron of Savernake Hospital in Wiltshire.

### BUSINESS AND SOCIAL AMENITIES AT "BART'S.'

At the summer meeting of the League of St. Bartholomew's Hospital Nurses, which took place last Saturday in the Great Hall, a scheme of post-graduation lectures was laid before the members by the president, Miss Isla Stewart. It is proposed to appoint a lecturer at a fixed salary, who of course will be a medical man. The admission of certain other nurses, who are not members of the League, on payment of 1s. for each lecture, was debated, but left over for decision until it had been further discussed in committee. After the business of the meeting was over the usual social gathering was held, with tea and music. It was a pleasant re-union of old friends, and the value and success of a "Bart's" training were sufficiently attested by the presence of the matrons of four other large London hospitals—namely, University College, the Royal Free, the New Hospital for Women, and the Royal Hospital for Diseases of the Chest. The children of the married members had been invited, and formed an additional attraction to the picturesque scene. Everyone was cordial and hospitable, and the matron looked well pleased to see her old workers gathered around her.

### THE MATRON OF THE NEW LEICESTER INFIRMARY.

An excellent appointment has been made by the Leicester Guardians. Having built a very handsome modern infirmary, they have selected as first matron, Miss A. B. Clarke, at present matron of Portsmouth Workhouse Infirmary. Miss Clarke having had fever training at Birmingham City Hospital, entered the Birmingham Infirmary for general training under Miss Gibson, and during the course of three years gained a first-class certificate. She also obtained a midwifery certificate. Leaving Birmingham she steadily advanced from sister to matron, and her excellent testimonials from the authorities of Portsmouth Infirmary, an institution with 700 beds, show that in the post she is now leaving she has acquitted herself with conspicuous success. The number of applications for the appointment at Leicester was very large, partly owing to the fact of the infirmary being new and partly to the salary offered being £130, rising to £150. The other candidates selected were Miss Orchard, matron of St. Olaves Infirmary, London, and Miss Dowbiggin, matron of Shirley Warren Infirmary, Southampton. Leicester Infirmary will not be opened until September, but Miss Clarke will take up her duties some little time before the opening.

### WOLVERHAMPTON VICTORIA NURSING INSTITUTION.

There was an excellent attendance of subscribers and friends at the annual meeting of the Wolverhampton Victoria Nursing Institution. The Mayor, in moving the adoption of the report, said that the institution holds a premier position in Wolverhampton, considering the boon which it confers upon the rich and the greater boon which it confers upon the poor. He alluded, of course, to its two branches of work—the private and the district. The accounts of the former show an increase of £158 in fees, and those of the latter a balance in hand of £46, so that both branches are in a satisfactory pecuniary condition. As to the work of the district nurses the number of cases treated in the year was 579. The matron of the institution, Miss Loveys, merits the many compliments which were paid to her by the Mayor and other speakers at the meeting.

### SOCIETY OF TRAINED MASSEUSES.

On Wednesday last week the members of the Incorporated Society of Trained Masseuses had the advantage of listening to a very interesting and practical lecture on "Deformities" kindly given by Mr. Muirhead Little, honorary surgeon to the Royal Orthopædic Hospital, etc., in the lecture-room of the Trained Nurses' Club, Buckingham Street, Strand. The lecturer dealt principally with affections of the spine, especially scoliosis, about which he said the masseuse could never hear and learn enough; also with torticollis, whether of spinal origin or not. Having touched lightly upon some of the commoner deformities of the lower limbs, he offered to give another lecture in the autumn in which he proposes to treat of these more fully. The room was crowded with an enthusiastic audience.

### The Nursing Outlook.

"From magnanimity, all fear above;
From nobler recompense, above applause,
Which owes to man's short outlook all its charm."

### SUPPLY AND DEMAND; PROBATIONERS.

THERE is one aspect of the large and increasing expenditure upon buildings in connection with the greater hospitals in this country during recent years which is apt to be lost sight of. Not only has the quality of the training of nurses and the educational standard of probationers greatly improved, but this expenditure upon buildings has provided much necessary accommodation of the best type, the absence of which made it impossible formerly to train probationers at all in proportion to the existing needs. So far as it is possible to estimate, it seems probable, taking the country as a whole, that the average supply of trained nurses is now capable of being maintained at a level, almost, if not quite equal to the demand which is likely to arise in normal years. The expense which the hospitals have incurred to enable them to train this adequate number of nurses entitles them to the gratitude of the public, and they should receive much more cordial recognition than has heretofore been accorded to the authorities of these institutions.

Another point worthy of consideration by those immediately interested in the training of nurses is the number of those who apply for training. Taking 500 hospitals and Poor-law infirmaries of 50 beds and upwards, with a few other nursing institutions, for 1904, as the basis, the yearly applications may roughly be estimated at 36,000. These 36,000 applicants include all who write for particulars and express a wish to be trained. It appears, however, in practice, at some of the best regulated nurse-training schools, that 50 per cent. of the applicants are rejected, for reasons shown, on their first letters alone. When the remaining applicants have been interviewed, 90 per cent. have been found to be unsuitable; so that in fact only 5 per cent. of the original applicants, where the test is exhaustive, may actually be received for preliminary training. These figures afford good reason for insisting on the importance of co-operation between all nurse-training schools, though it is fair to conclude from the large number of applicants and of those rejected, that the same women must apply over and over again. That is to say, those rejected at one hospital may send in an application to hospital after hospital, and thus some of them may succeed in ultimately securing admission, somewhere, for preliminary training. Now, an applicant may or may not be suitable, but it is right to infer that no well-organised training school would reject applicants except for good cause. Assuming this to be the case, it would save the authorities of all the schools some expense and much time and vexation if they would co-operate to the extent of keeping each other informed as to what their decision has been in regard to original applicants for training. Where the utmost care was taken to exclude unsuitable women it was found that quite 11 per cent. of those entered for preliminary training disappeared at the end of the three months' trial. Hence, where less care is exercised the percentage of disappearances must be infinitely greater. It ought to be possible for the nurse-training schools in London, for instance, through their matrons, to co-operate in this matter to their mutual advantage. In any case care should be taken, by exacting payment for preliminary training, to free the hospitals from all expenditure under this head.

In the course of the evidence taken by the Committee of the House of Commons some witnesses have stated that there would be no hardship if the training schools refused to pay their probationers. The contention was that the payments are so small as to be of no practical value to the probationers. Evidence of this kind appears to us to be without knowledge, and to show an absence of apprehension of the circumstances of the majority of those who at present offer themselves for training as nurses. We believe that the small salaries given by the training schools are of vital importance to the majority of probationers and their friends. Very often these possess no means of their own; the resources of their friends are small; and many women, who now find congenial employment in the nursing world, would never have been permitted to enter as probationers, were it not for the fact, that the hospitals pay some salary during the period of training.

We may reiterate that in practice not more than six per cent. of the actual applicants each year are received for training. In five hundred institutions the yearly average vacancies amount to about 2,100, that is, to a number which is less than six per cent. of the total applicants. These figures enforce the lesson that co-operation between the nurse-training schools, with a view to establish a system whereby the same unsuitable applicant may be prevented from putting a number of schools each year to the trouble of rejecting her over and over again, is highly advisable. It is undesirable in every way to permit the possibility of an unsuitable candidate obtaining admission to preliminary training, with all the consequent trouble and expense entailed, from any laxness in the existing system. So much work results to the schools from the training of nurses, that each authority should welcome any step which is calculated to lessen its volume, and to prevent the waste and loss of time which result from the present absence of co-operation in regard to the supply of probationer nurses.

### The Nursing of Sick Children.

By James Burnet, M.A., M.B., M.R.C.P.Edin., Registrar, Royal Hospital for Sick Children; Clinical Tutor, Extramural Wards, Royal Infirmary; and Physician to the Marshall Street Dispensary, Edinburgh.

### XIII.—DISEASES OF THE EYE, EAR, NOSE, AND THROAT.

In this, our concluding lecture, there is much to be discussed, and accordingly we must be brief and somewhat pointed in our remarks. First of all let us look at

### Some of the Commoner Eye Affections of Childhood.

There is a disease met with in newly-born infants known as ophthalmia neonatorum. This is really a severe inflammation of the mucous membrane of the eye associated with pus formation. It must always be remembered that this is one of the most serious of all eye diseases, and if not promptly and carefully treated will inevitably produce total blindness. The nurse's duty in such cases is not only to carry out the orders of the medical attendant most thoroughly, but also to guard against personal infection. In these cases the inner surface of the eyelids is usually brushed over with some silver preparation. This part of the treatment is generally carried out by the medical attendant himself. In addition, however, the nurse has to douche out the eyes every hour with a warm antiseptic lotion. As this is not always an easy matter, a few general hints on the subject may not be amiss, and these may be taken as applicable to all cases in which douching of the eyes forms an essential part of the treatment.

*How to Douche the Eyes.*—A glass syringe is first filled with the warm lotion. The patient is firmly held in a blanket with only the head exposed, and he should preferably be laid on a couch with the head supported on a pillow. The eyelids are then separated, and the nozzle of the syringe introduced at one corner of the eye. The fluid is then slowly made to flow over the surface of the eye, and in so doing it washes away the pus. Every now and again the nurse should mop out the eye with absorbent wool, which has previously been wrung out of the lotion. When all the pus has been got rid of the lids are carefully dried with cotton wool and their edges gently rubbed over with a small piece of weak yellow oxide of mercury ointment. In most instances, where assistance is obtainable, the fluid as it trickles from the eye may be collected in a basin held against the patient's cheek; but this may be dispensed with if a mackintosh sheet is pinned round the infant's neck.

Inflammation of the eyelids, technically known as blepharitis, is a very common condition. Crusts form on the edges of the lids, and if these crusts are not carefully removed by soaking with warm water or olive oil the eyelashes are apt to fall out, and a condition of blear-eye result. Sometimes ulcers form upon the surface of the eye, and these are always more or less painful and associated with intense fear of the light, so that the patient keeps his head buried in the pillows or under the bed-clothes. They are very frequently met with amongst children of the poorer class, whose home surroundings are dark and unhealthy and whose food is not of the best. Accordingly, all such cases usually derive great benefit from tonic treatment and a nutritious diet in addition to local applications.

### Ear Affections in Children.

Pain in the ear, or earache, is of such frequent occurrence that we may mention it first. Its cause is extremely variable. Sometimes it is reflex from the irritation of a tooth; in other cases it is due to the presence of hardened wax. If associated with fever and vomiting, it may indicate grave ear disease. In every case the throat should be carefully examined, as inflammatory conditions of the tonsils readily produce earache. Its treatment is usually most satisfactorily carried out by the application of heat. The nurse may apply a bag of hot salt over the ear, or she may drop in a small quantity of warm oil. The use of other remedies must be left to the physician himself.

Discharge from the ear, or otorrhœa, as it is usually termed, is very common after measles and scarlet fever, as well as in connection with certain other diseases. Whenever there is this condition present it becomes necessary to syringe the affected ear at regular intervals. The mode of syringing the ear is as follows: A teaspoonful of boracic acid crystals is dissolved in a breakfast-cupful of warm water. This solution is run into the ear by means of a glass syringe having a bulbous end. The patient should have a mackintosh sheet pinned round his neck, and a kidney-shaped basin should be held at the side to catch the returning fluid. While injecting the lotion the nurse should with finger and thumb pull the lobe of the ear upwards and backwards, as by this means the canal is made to form a straight tube. When the syringing is completed the nurse should carefully dry out the ear with a piece of cotton-wool twisted into a spiral. As a rule it is not advisable to place any wool in the ear after the operation is completed, as this tends to dam back the discharge.

Sometimes small boils form within the ear, and these are exquisitely painful. Their presence in young children is apt to be overlooked. Eczema may affect the ear, and is very apt to be present in patients suffering from a discharge which has been neglected. In all such cases scrupulous cleanliness must be observed by the nurse, who should carefully dry the ear, not only after syringing, but whenever it becomes wet with discharge.

### The Nose and Some of its Affections.

A discharge from the nose is a condition met with under quite a variety of conditions. It may be merely symptomatic of a simple cold, or it may indicate the presence of a foreign body. Again, it may be significant of serious constitutional disease, especially if met with in infancy and associated with snuffling and skin eruptions. In particular every nurse should remember that a discharge from the nose of a purulent character may be the only indication of diphtheria present, and accordingly she should be on her guard not only against the possibility of the spread of the infection, but also against delay in consulting a competent medical adviser.

Epistaxis, or bleeding from the nose, is rarely a serious condition in children, though it may be symptomatic of grave organic disease in some instances. In cases of epistaxis the child's head should be raised and pressure applied over the sides of the nose. Failing this, the nostrils may be plugged with cotton wool soaked in hazeline.

In febrile conditions the nurse should pay strict attention to the condition of the nostrils, as these are apt to become blocked up with crusts of hardened mucus, which may not only give rise to a disagreeable sniffling, but even to interference with nasal respiration. Whenever these crusts begin to collect the nurse should soften them by smearing a little vaseline inside the nostrils, and then gently remove them with a pair of dressing forceps. The relief afforded to patients by attention to this apparently trivial matter is often very marked.

In some conditions it becomes necessary to syringe the nose, and this is best carried out by using a syphon tube. A simpler plan, however, is to lay the patient on his back, and then slowly to pour the solution from an ordinary teaspoon into the nostrils, or, if preferred, a medicine dropper may be employed instead.

### The Throat and its Diseases.

Every nurse should remember that a slight sore throat will often send a child's temperature up to 103°, or even 104°. Then, again, sore throat may be the initial symptom of scarlet fever, of diphtheria, or even of some of the other infectious fevers. Affections of the tonsils are of very frequent occurrence in children, and in some instances tonsillar inflammation may signify that the child is suffering from rheumatism. Enlarged tonsils, either with or without the presence of adenoids, give rise to a group of symptoms, such as snoring at night, mouth breathing, deafness, and chronic cough, the real cause of which may be overlooked. From what has been said it will readily be gathered that affections of the throat in children are of the very first importance.

Diseases of the throat are usually most successfully treated by the application of pigments of various kinds. Gargles are of no use whatever, as even if the child could use them, they seldom if ever reach the affected parts. Painting the throat of a child is usually a very difficult and trying task for the nurse. In doing so the child's head should be firmly held by an assistant, while the nurse, depressing the patient's tongue by means of the handle of a spoon, passes the brush well back, and then sweeps it rapidly over the affected area. On the whole a straight brush is to be preferred to one which is bent at an angle, as the latter is not only more difficult to use, but is very liable to get broken during the process of painting.

We have tried to give a very brief outline of the various subjects embraced by the title of this lecture. Space forbids further consideration of many interesting facts connected with them. We trust, however, that this and the preceding lectures will aid nurses not a little in the better performance of their duties, and if they lead those who peruse them to seek for further knowledge our efforts will not go unrewarded.

### The Nurses' Clinic.

### HOME-MADE APPLIANCES. BY A SUPERINTENDENT OF DISTRICT NURSING.

Arriving late one evening at a house three miles distant from the Home, I was greeted by the patient with, "Nurse, the doctor says you are to give me a vapour-bath to-night." I knew that the district cupboard contained wicker cradles, a spirit-lamp, a bronchitis-kettle, and mackintoshes. The husband offered to go and fetch them, and a note was written to the nurse on duty telling her what to send.

While waiting for the appliances I got everything ready as far as possible. I placed a large kettle of water on the fire, sent out for methylated spirits to replenish the lamp, and collected all the blankets that could be found. I took the patient's temperature, pulse, and respiration, and wrote the report.

Presently the husband came back with the news that the appliances for the vapour-bath had been sent out already to another case. It is an unwritten law that any order given by a doctor must be carried out the same day, so I was obliged to see what could be done with home-made apparatus. I protected the mattress with some old sacks and brown paper, and placed a blanket over this. I sent for several yards of cane and tied it in three arches across the bed. I took an ordinary tin kettle, half-filled it with boiling water, and lengthened the spout with a peashooter and part of a tin whistle. A spirit-lamp was bought for sixpence. I put the American-cloth table-cover over the cane hoops, and covered all in with six blankets. I hung the bath thermometer on one of the cane hoops, and again took the patient's temperature.

I then lighted the lamp and raised the air inside the cradle to 130°. When the patient had been in the bath 10 minutes I took her temperature, and carefully observed if she perspired in the axillæ. At the end of 20 minutes I put out the lamp and again took her temperature. I let her rest for about 10 minutes, wiped her with warm towels, put on a warm dry nightdress, covered her with a warm dry blanket, and gave her some hot milk to drink. The patient had chronic rheumatism, and the bath, repeated every night for many weeks, gave great relief.

A vapour bath can also be arranged by taking a small clothes horse instead of cradles, and covering it with stout brown paper and blankets. Bring the bed up close to the fire, and add a spout of brown paper to an ordinary kettle.

A *Hot-air Bath* can easily be given. A small bedroom lamp with a glass (price 6½d.) should be placed on an old kitchen plate and protected by a bread grater (price 4d.). The lamp must not be turned high, and must be carefully watched. A thermometer must be hung up inside the cradle, and the air may be raised to a temperature of 150°. When the patient has perspired freely he should be rubbed with hot towels and have on a dry warm nightshirt, be given a hot drink and be well covered up.

*Vapour Bath on Chair.*—If it is not possible to give the bath in bed, take a cane chair, place a blanket on it, letting it fall fourfold over the front legs. Place a flat pan containing boiling water under the chair. Seat the patient on the chair with his feet on a stool, and place a blanket round the patient's neck and completely covering him and the chair. Keep the temperature of the water up by adding bricks that have been heated in the oven, or placed on a trivet over the fire, or stood on a closed stove. The patient must remain in the bath until he perspires freely. If there is an old-fashioned round sponge-bath in the house the chair can be placed in the middle, covered with a small blanket, and the patient seated on it with his feet on a wooden footstool. A large blanket must be fastened round his neck, and hang over the edge of the bath, making a kind of bell tent. The water can then be poured into the bath, and the heat kept up by adding hot bricks. A hot-air bath can also be arranged on the same principle. A small lamp must be placed on an old plate and protected by a bread-grater, and the hot air directed under the chair.

*Screen to Keep off Flies.*—A sailor suffering from typhoid fever and left much alone, was greatly annoyed by flies, which he was too weak to brush away. We sent to the toy-shop for a long piece of cane and fastened it on to the head of the bed, bending it forward in a curve. The wife produced an old white muslin curtain, we draped it over the cane, and a most effective screen was made. A small curtain should be arranged in the same way to protect infants and young children when sleeping in the daytime in summer weather. In order to interfere as little as possible with the supply of air the screen must be of very coarse muslin or of netting with a small mesh.

*Bed Pulleys* can either be made of 6 feet strips of knitting from 2 to 5 inches wide (according to weight of patient) or of strips of unbleached calico, stitched together by machine, or of rope (½d. a yard), well padded with tow and clean rag sewn round at the parts where the patient grasps it.

*Bed Pans.*—Wooden rims can be cut to fit ordinary enamel basins or deep pie dishes.

*Bed Table.*—A very good bed table for convalescents can be made of a piece of ¾-inch deal, about the size of an ordinary pastry board, slightly hollowed out in front, and with a leg 9 inches high fixed at each corner. If the patient needs to lean heavily forward the table is better made of a packing case with two of the sides removed except for about 3 inches at the corners.

*Bed Pockets*, to hold the invalid's handkerchief and other little necessaries, may be made of washing print, and should be about 16 inches by 12 inches with strings run round the top. The bag can be hung on the bed rail or fastened with a safety pin to the counterpane.

*Pillows.*—If extra pillows are required they may be filled with oat chaff, which can be obtained cheaply at any seed merchant's or from livery stables. All pillows must have double covers, so that they can easily be kept clean.

*Hot-Water Bottles* may be made of empty spirit or vinegar jars, and must be very securely corked and wrapped in flannel or baize. In very poor households a firebrick (price 1½d.) heated in the oven or on the stove and well wrapped up in flannel will be found an easier means of warming the bed.

*Feeder.*—A small earthenware teapot makes a good feeder. The spout must be carefully cleaned several times a day with a feather or a pipe-cleaner.

*Screens.*—A clothes-horse covered with an old cotton counterpane makes a good screen, and enables the window to be widely opened even in small rooms without exposing the patient to draughts.

*Ventilator.*—In cases where it is advisable that, in addition to the use of a Hinckes-Bird ventilator, the window should be left permanently open at the top, a strip of butter muslin, or any similar material, should be nailed across. This will strain out flies, dust, and damp, and break the air up into very small currents.

### Incidents in a Nurse's Life.

**Contributions for this column should be addressed to the Editor, and if accepted will be paid for.**

### A MORNING IN THE MEDICAL OUT-PATIENT DEPARTMENT OF A SCOTTISH CHILDREN'S HOSPITAL.

Our out-patient department, which is quite new and beautifully fitted up, is built apart from the hospital. The medical portion of this department consists of an outer waiting-room, where the names of new patients and the cards of old ones are taken; an inner waiting-room, where the little patients are undressed by their mothers preparatory to being examined by the doctor; a vaccination-room, where vaccinations are done two days in the week; two isolation-rooms, in case of children with infectious diseases coming up by mistake for examination; a dispensary, where the patients get their medicine free, only being required to pay one penny for the bottle, unless they have brought a bottle with them. A donation box is always at the dispensary window for those who can afford and are willing to give a small contribution.

Our morning begins, of course, with the necessary dusting, etc., by the probationer, the setting of things in order for the doctor—register-book, papers, cards, and any medical appliances, etc., which are likely to be required during the consulting hours.

The patients, with their mothers, begin to gather in the outer waiting-room from about 10.30 a.m. The names, addresses, and ages of the new patients are taken; these are entered in the book by the Registrar; a prescription form and a card with the patient's name and number, according to the register, are made out. The patients are then shown into the inner waiting room, where each mother is instructed to undress her child and wrap it in a blanket, which is provided for the purpose. In turn they are called into the consulting-room. Each patient's mother is given a card, with the number of patient's prescription form, which must be brought on each visit. Some of the mothers are very careless, and either lose their cards or forget to bring them when they come. This gives us endless bother looking for the names in the register, so as to find the number of their prescription. We always do our best to impress on the mothers the importance of taking care of their cards; but, in spite of all, some of them lose them "in the flitting," etc., or "forget to bring them up, and it's too far to go back again." These are some of the common excuses. It is also quite a usual thing for them to "leave them in the car" or "drop them on the way up," Then they are never quite sure when they were up at first, which makes it all the more difficult to find their names.

On the other hand, there are always the careful mothers, who never come without their cards. Our out-patient doctor (we have three different doctors, each coming two days a week) begins work at 11 a.m. The mother of the patient, with the child on her knee, sits on a chair facing the doctor, who makes his examination and the necessary inquiries, and writes his notes accordingly; makes his diagnosis, gives the mother instructions as to treatment, feeding, etc., and writes a prescription if he thinks it necessary. The patient is then taken out of the consulting-room and another comes forward, and so on.

As each prescription is written it is handed to the dispenser. The patients who have seen the doctor wait at the dispensary until their medicine is ready.

The doctors' examinations as a rule last until after 1 p.m., and on busy days it is sometimes 2 p.m. before the consulting room is cleared of patients. Those cases which the doctors consider requiring special attention are sent into the wards of the hospital, the mothers of the others are given instructions as regards treatment, feeding, etc., and are told to bring the children back in a week or more as may be necessary. During the doctor's consulting hours nurse and I are kept very busy. Besides bringing the women into the consulting room, in order, with their children, there are numerous ways in which we require to assist the doctor, temperatures to take, children to weigh, etc. No patients of over 12 years are seen at our hospital. Out-patient work becomes very interesting when one gets to know the children as they come up from time to time. It is nice to watch the progress of the little ones whose mothers carefully carry out the doctor's instructions. There are always the careless ones who seem to forget and don't do as they are told; then they wonder why their children do not get on. We see some very sad cases who come from such dirty miserable homes. Our average attendance per week is about 200.

### The Nursing Department in Paris Hospitals.

BY A CORRESPONDENT.

Already rising high in the wide-spread grounds of La Salpêtrière is a building destined to confer upon the "infirmarian" of the Paris hospitals the status and education of the trained nurse. Schemes of instruction for "infirmarians" have, it is true, been in existence ever since they themselves began in 1880 to take the place of nuns in attendance on the sick. Yet little if any progress has been made in raising their status during the last 25 years, so fruitful in nursing progress here and in other countries, and this for several reasons. In the first place, duties towards the patient have held no higher place in the scheme of things than the general duties of the institution; skilled and unskilled work have not been discriminated, and the coarsest labour must alternate with delicate medical and surgical processes. Next, the pay has been low, the hours long, and the duties, owing to the insufficient numbers of the staff, arduous in the extreme. We do not believe that any of these reasons would have deterred the right sort of woman from devoting herself to the care of the sick poor. They certainly proved no bar to the pioneers of nursing in this country. But, truth to tell, the conditions of life imposed on the "infirmarian" are prohibitive. The Director-General of the Assistance Publique, in pleading for a reform, gave the following description of the state of things which existed only a short time ago:

"After days of work, lasting sometimes from 14 to 15 hours, repose is barely possible in dormitories difficult to classify, where the beds touch, where air is wanting, where it freezes in winter, and where in summer the heat is intolerable. As you know, there are no lavatories; there are no water-closets: all must live in a promiscuity harmful to morals and tending to stifle all sense of personal dignity."

"There is nothing to retain the attendants of either sex in the institution of which they make a part. They are not at home; they feel they are almost strangers, if not pariahs. They possess neither private abode nor common sitting-room, and no recreation of an innocent nature is open to them."

Many of the "infirmarians" are men; some are married women, living out of the institution. There is no matron, no supervision except what is exercised in each ward by the "surveillante"—herself promoted from the ranks—and what is most unfortunate of all, no power in any institution of altering a condition of things known to be faulty. It is not surprising that lectures, however able, twice a week, should prove inadequate to produce a trained staff under these conditions. No limit can be placed on possible endurance where reform may be achieved. But when reform is barred, devotion is discouraged or turned into another direction.

Under the new scheme, to be gradually introduced, the male "infirmarian" will disappear except for attendance in special wards. All probationers received for training will be required to pass an elementary examination in reading, writing, and arithmetic, and will be under instruction in a special training school for a period of two years, during which time they will attend lectures and receive practical class instruction in nursing outside their ward work. At the end of their training they will be required to pass an examination, and may then receive a diploma as 'infirmière." There are four classes of "infirmières," each subject to an increase of salary, and it is necessary to pass through these grades before being appointed "surveillante," a position answering in some degree to that of "sister." There are, again, four grades of "surveillante," the highest salary rising to £48, with an allowance of about as much again should the surveillante prefer to live out of the institution. There have been misgivings on the part of those in authority with regard to creating the post of matron, as it is thought it may conflict with the authority of the director, who in each Paris hospital is responsible for the control. There is, however, to be a senior surveillante responsible for keeping order among the nursing staff. This senior sister will not herself be entrusted with disciplinary powers, but she is charged with the duty of bringing cases of misconduct before the director. Grave cases of misconduct calling for fines, loss of promotion, or dismissal, must be referred to the Council of Discipline of the central administration. It is expected that the first training school will be in working order by the middle of next year. To achieve all that is hoped for it in the inauguration of a new era of trained nursing in the Paris hospitals two conditions should be fulfilled. A gentlewoman, herself a highly-trained hospital nurse, familiar with administrative work, should be placed in a position of authority over the pupils, and a period of at least two years' residence should be required from probationers. It is not clear that the authorities realise that, however excellent the teaching, a period of residence is indispensable, nor that they understand the value of the influence exercised by a good matron in the training of her nurses. It is not after all the comfortable quarters, nor even the admirable teaching, which have gone to make the great English training schools celebrated throughout the world. It is the spirit of devotion to duty which in a well managed hospital spreads impalpably down from the highest to the lowest, where uninterrupted by the claims of home life the probationer becomes conformed to the demands of discipline. It must not be expected that instruction, however careful, administered in hourly classes to day pupils will make a nurse, or that a woman, however highly qualified, who leaves her own home day by day to superintend a school of probationers, can have the same effect in moulding character as one who lives with them and shares their life.

The provisions for regulating the new school of nursing have not yet been crystallised. It is permissible then to express the hope that the "externe" part of the system may be regarded as an evil necessary only at the very commencement, and that it may finally disappear altogether.

It will have been seen from articles which appeared recently in our columns on the nursing question in Paris and Bordeaux that public opinion in France is awaking to the value of good nursing, and that the movement in favour of reform initiated by the late M. Mourier is being vigorously forwarded by the present Director-General of the Assistance Publique, M. Mesureur.

### Nursing the Schoolboy.

BY THE MATRON OF DENSTONE COLLEGE SANATORIUM.

Boys as I Know Them.

After many years' experience in general nursing, which includes six years specially devoted to the nursing of schoolboys, it has occurred to me that a short account, written in simple language, may be of some use to those who take up the work of matron or nurse in our large public schools. In schools where there is no separate sanatorium it is, of course, an advantage that the matron, whose duties include the charge of the health of the boys, should be a trained nurse, but "professionalism" in school nursing ought, I think, to be conspicuous by its absence. The training is, of course, most useful in cases of acute illnesses, and in accidents where surgical dressings are required, but I would rather trust a boy to a sensible motherly woman, with nursing instincts and experience in the management of boys, than to the most highly trained professional nurse who lacks other most essential qualifications. It is impossible to insist too strongly on the need of the motherly instinct in a school matron. She should be one who is capable of feeling that, for the time being, "the boys are her own"—a sentiment which, strangely enough, comes naturally, and cannot be acquired even by those who are mothers themselves. Also, she should be contented, at any rate during term time, simply "to live for the school." Without these qualifications I do not think anyone, however highly trained, can be a really successful nurse for boys; with them, it is impossible to find a happier or more useful occupation.

For a woman without near ties of her own I think the life is ideal; it is full of variety. No two boys are alike, and to dwell for three parts of the year in the midst of two or three hundred bright, joyous young lives is the best antidote, in my opinion, against developing morbid symptoms in middle life. Moreover, anyone with fairly good health ought not to be considered "too old" for such a post, even when on the wrong side of forty.

Boys are responsive, affectionate, and grateful; they keenly appreciate personal service themselves, and are always generous in letting their people know that they are well cared for in the "San." They are, I find, when in the sanatorium utterly devoid of selfishness; they never grudge any kindness or attention they can bestow on a comrade, and are entirely free from petty jealousies. In convalescence I find them most willing helpers, obedient, polite, and thoughtful. Every detail of a schoolboy's life is full of interest to one who loves them: the choir-boys with their fresh young voices, their reverent behaviour in chapel, school Confirmations, a boy's first Communion, the stillness of the playing fields during school hours, the joyous exuberance when the school is "let out," the keen competition in games, the friendly rivalry between houses, sports days, Speech day, the play, and, what perhaps appeals to me most of all as a nurse, their splendid patience and absolute trustfulness in serious illness.

I do not wish to imply by what I have said that the average schoolboy is a saint—far from it; and I would not have him other than he is. Mischief is dear to him, chaff and sarcasm flow freely, yet there is seldom heard a spiteful remark, and, if one will only look for it, there can be found the germ from which springs the heroism, the manliness, the unselfishness, which in days of darkness and emergency have characterised all over the world the men trained in the public schools of England.

The Sanatorium.

An ideal sanatorium has the conveniences of a modern hospital with the surroundings and comfort of home. I feel it is the greatest compliment a boy can pay to the school and to me when he says, "It is like going home to come here."

For a school of 300 boys a sanatorium capable of accommodating 30 to 40 inmates ought to be sufficient; it should, if possible, be ground floor only, arranged with exits into two distinct sections, each with its own bathrooms and lavatories, for the purpose of isolation during infection. Each section should contain one large ward for 10 or 12 beds, a couple of small wards for two or three beds each, a good dining-room, and a convalescent room.

The large rooms, corridors, and bathrooms should be heated by radiators, but there should also be an open fireplace in each room; the well firegrate is, I think, the most economical and cleanly. The walls should be painted in soft greys or greens, the woodwork white, the floors mosaic, with a cork mat beside each bed. The curtains of linen should tone with the walls; and the furniture should be of as light a description as possible, and of a nature that can be easily cleaned and disinfected. Basket and American folding-chairs, with loose cushions covered with a pretty cretonne, I find the best; screens of plain wood or bamboo, with Turkey-red curtains, and a little red introduced into the tablecloth, give a little warmth to the appearance of a large ward. Each bed should have what is known in hospital as a locker, for dressing-gown and slippers, &c., and a small drawer for letters and personal belongings, the top of which, covered with a pretty cloth, serves as a table; but of course adjustable bed-tables and small bamboo ones are very useful; and there should always be a few cut flowers and plants, which in the sleeping-rooms would be removed at night.

The more dainty, comfortable, and home-like a sanatorium is made, the more the boys will value it; and I find them most ready to subscribe to an "embellishment fund," which comes in useful for flowers, pictures, easy chairs, or any luxury which the college may not feel justified in supplying. With the daily average of four boys during last year not a single article was destroyed or misused.

If possible, I think that the domestic and culinary department should be distinct from the school and under the management of the nurse in charge; but this need not be an expensive arrangement if the nurse possesses some little idea of household management and cooking. If she does not, it is an excellent opportunity for acquiring much useful knowledge. The household must necessarily consist of at least two persons, the nurse and one servant; and I find that in a school of 300 boys the average number of boys on sick-leave from school daily is about three in the winter and two in the summer term, and the cost per head does not exceed 8d. per day. Of course, during an epidemic the numbers would be increased, and the cost per head would be slightly less.

Only acute cases need special diet. I give my convalescent boys for breakfast coffee made with three parts of milk, an egg, marmalade, and a liberal supply of butter on their bread. At 11 a.m. they each have half a pint of milk and two or three biscuits; for dinner, meat, vegetables, a milk pudding, or one equally wholesome and digestible. As a foundation for all my puddings not made with milk only, I use breadcrumbs made from stale pieces of bread, in preference to flour, with a little sago, and carbonate of soda instead of eggs. For tea they have occasionally buttered toast, but as a rule bread-and-butter and jam; for supper they can have porridge, bread-and-milk, cocoa, bread-and-butter and biscuits. I always have plenty of good soup, the stock for which I make of uncooked bones, after removing every particle of fat—using the latter for plain cakes.

I thicken the soup with cornflour mixed with milk, add salt to taste, and a little browning. When I want it to be extra good I add two teaspoonfuls of Bovril to every pint. The soup is for those who cannot take meat for dinner, as well as for lunch and supper for such as cannot take milk or need extra "feeding up."

I never have any complaints about the food, and the boys always seem to enjoy it. All boys who are well enough get up in time to have breakfast with me, and I always dine with them. If a boy is specially delicate or recovering from a severe illness, he is allowed to be what is termed "a lodger," namely, he attends school, but comes to the sanatorium for his meals and sleeps there. I think this is a great advantage to him, as he is not expected to be present at early school or chapel before breakfast.

(*To be continued.*)

### A Hospital Tragedy.

BY AN EYE-WITNESS.

We were very busy in the typhoid ward when one afternoon the ambulance brought us a fresh patient, an old man 65 years of age. We had great difficulty in undressing him, for he was very ill and quite delirious, and if left for a moment would keep jumping upright, which as everyone knows, is almost fatal in typhoid fever. We got him to bed and the resident medical officer saw him, looked very grave, and said to the senior nurse, "Keep a keen watch on him; there is danger of perforation."

However, two weeks went by, and still the old man was with us. His delirium had ceased and indeed he was one of our most placid patients. For long hours he would lie looking through the opposite window where nothing could be seen but green field and sky. All through his delirium he had raved over someone named "Bob." Bitter curses always followed the name, then a fit of weeping would come on, and he would clutch at a nurse's arm and ask, "Is he dead? tell me he is dead;" and to pacify him she would say Bob was dead, and his answer almost invariably was: "Then he has got his deserts."

He would then settle down for a short time till another paroxysm came on. He had a serious relapse some days after his crisis had been passed, and was given up by all the doctors, when to everyone's astonishment he rallied again. Nearly every day the matron would remark, "I don't think we shall lose him, after all"; and we did not.

He had progressed so far as to be able to sit up in bed, with a bed-rest, when one day a new patient was brought in, a man nearly 30 years of age, his face bronzed and lined, as though he had been abroad—a soldier, perhaps. He was very ill, for he had pneumonia as well as typhoid, so it happened that he was screened off from the view of the other patients whose beds were opposite, as was that of the old man. From the time of the arrival of the new "case" the old man was restless and always anxious to see what manner of man was behind the screen. This seemed strange to us, as he had never before evinced any curiosity about other patients. Two or three days passed and our new patient gradually became worse, until it just seemed as though it could only be a matter of a day or two at most for him to live. In the morning of the day he died the old man solemnly told the night nurse who gave him his breakfast that he had been dreaming all night of his son, whom he had not seen for 11 years. He declared that he had heard his son's voice, and he also added, very quietly, "My revenge is at hand! I know it! I feel it is near!" The nurse suspected that his mind was a bit disturbed by dreaming, as he was still very weak, so she thought no more of his words until afterwards, when they came back to her mind with a terrible significance.

The same afternoon the visiting physician came with a few students, and they were for some time at the bedside of the dying man, when, to give more light, our own resident doctor pulled aside a screen. The next moment, a most fearful scream broke from the lips of the old man, "Bob! Bob! you here! Curse you!" and a great many more imprecations. A nurse tried to quieten him, but with abnormal strength he pushed her away, and stood up in bed. "He is, or was, my son"! he shouted—"I will see him"!

One of the doctors came forward, and placing his hand on the excited old man's shoulder, said kindly:—"Calm yourself, and if you are his father, nurse shall wrap a dressing-gown round you, and you may come over to see him; but only on condition that you are quiet, for the man over there," nodding his head towards the screen, "is dying, and if you have a wrong against him, your judgment is taken out of your hands, for he will soon be called to his account." While he was speaking, the nurse had fetched some clothing to wrap the old man in, who was trembling with suppressed excitement, and kept muttering all the time:—"I knew it, I felt it, and to think he has been here for days, and I've never told him!"

He was helped across to his son's bedside, and the medical men moved aside to make room for him.

"Bob!" he exclaimed in stentorian tones. The dying man, whose now feeble brain was the only part of him which seemed alive, opened his eyes. He moved as though he would have liked to have put his hand out.

"Dad, forgive," he whispered, but the old man, whose weakness seemed to have fallen from him like a cloak, stood upright, splendid in his wrath.

"Never! as long as there is breath in my body, you craven. You killed your mother!"

Here our resident interposed, "Forgive! the man is dying fast," he said.

"Let him die—let him die!" and with a wild laugh the old man fell back in a swoon. Fortunately the doctor who stood near was able to catch him, or his head most certainly would have caught the edge of the stove. We got him back to bed. Tears filled the eyes of the dying man, and motioning with his hand to our resident he whispered: "I have atoned, I have suffered; Dad doesn't know."

Later on in the evening he passed quietly away, and the old man was kept under the influence of a sleeping draught to avoid another scene disturbing the last moments of the man "Bob." The next day, when the father knew his son was dead, a settled calm came over him, and never left him again until the day of his discharge. He went out restored to health—the old man whom no one thought would recover, whilst the other, the man "Bob," was cut down in his prime.

Was it the survival of the fittest? We wondered.

### To Nurses.

**We invite contributions from any of our readers, and shall be glad to pay for "Notes on News from the Nursing World," or for articles describing nursing experiences at home or abroad dealing with any nursing question from an original point of view, according to length. The minimum payment is 5s. Contributions on topical subjects are specially welcome. Notices of appointments, letters, entertainments, presentations, and deaths are not paid for, but we are always glad to receive them. All rejected manuscripts are returned in due course, and all payments for manuscripts used are made as early as possible after th beginning of each quarter.**

### Select Committee of the House of Commons on Nursing.

Evidence of Dr. Ernest White.

The first witness examined by the House of Commons Select Committee on Registration of Nurses on Tuesday was Dr. Ernest White, Professor of Psychological Medicine at King's College, London, and President of the South-Eastern Division of the Medico-Psychological Association, on whose behalf he appeared. Dr. White explained that the Association had been formed in 1841, with the object of promoting knowledge of the treatment of mental diseases. In 1889 a scheme was started, which had been but slightly altered since, for the examination and registration of mental nurses. Every candidate must have had three years' training in a recognised institution, or in not more than two such institutions. Originally the training required had been two years, but this had been extended to three. Besides public asylums, licensed houses for the insane, with usually not less than 40 beds, were recognised by the Association as fit places for training. The Association thought it advisable that part of the training should be in general nursing, which could be obtained in the sick wards attached to the asylums, but they had not been able to insist upon it.

The examination was held twice a year. The written part was conducted by three examiners, who were paid half a crown per person, each paper being read by only one examiner, while the *viva voce* part was conducted by the superintendent of the institution to which the nurse belonged and an approved assessor from some neighbouring institution. The number of candidates was three to four hundred at each examination, and the fee was five shillings, which covered all expenses, as the work of their registration board and registrarship was honorary. The number of candidates for the examination of the Association so far had been 6,500, or about a third of the total of mental nurses, and 75 per cent. had passed the examination. Though he would not say that their certificate was a *sine qua non*, yet it was almost impossible for a nurse to gain promotion if she did not possess it. He thought that there should be in any State registration scheme, four classes of nurses: (1) hospital, (2) obstetrical, (3) mental, (4) rural, the last constituting a lower grade of hospital nurses, and that the examination of mental nurses might well be delegated to their Association. Asked by a member of the committee he said he thought that it was necessary to have a uniform minimum standard of efficiency, and that this could only be attained by a central board, and not through examinations set by institutions. He deprecated a too high fee being charged if State registration were enforced, and thought that £1 would be sufficient.

Evidence of Dr. Shuttleworth.

The next witness was Dr. Shuttleworth, honorary secretary of the Asylum Workers' Association, who stated that the members of that body were encouraged to obtain the certificate of the Medico-Psychological Association, which they regarded in the light of a qualifying body, and submitted that it was important for the protection of the public that the class of private mental nurses should be considered in any scheme of registration.

Evidence of Mr. Patten MacDougall.

The evidence of Mr. J. Patten MacDougall, vice-president of the Local Government Board for Scotland, was then heard. He appeared in order to explain the system of registering of nurses, which had been adopted by the board for nursing in the Poor-law infirmaries. Mr. Patten MacDougall went into the history of the scheme, explaining that it had been originated in order to put a stop to the system of nursing of sick paupers by other inmates of the Poor-law houses. In order to induce the authorities to give up this system the Board gave a grant of half the nurse's salary and 3s. a week for every nurse who was qualified to be placed on the register of the Board. In 1889 the conditions of registration were stereotyped, and the Board was still tied by them, but the Report of the Departmental Committee showed many important changes, which were now advocated—notably a three years' training instead of the present two. The registration only applied to Poor-law nurses, and any nurse was entitled to registration if she had had two years' training and had passed the examination at an institution where there was a resident medical officer and was well reported by the matron. Mr. Patten MacDougall showed the Committee the actual Register of the Board, which gave the career of a nurse throughout the period she was nursing under the Board, though it did not take cognisance of work done after she left their service unless she wished to return to it.

The Committee will meet again on July 6th, but as there is no more evidence to be taken, the sitting will be held with closed doors.

### The Medical Practitioners' Association and the Registration of Nurses.

At the annual dinner of the Incorporated Medical Practitioners' Association last week, Dr. A. Rivers-Wilson, of Oxford, in the chair, the question of the registration of nurses was raised.

Dr. Ambrose, M.P., in proposing the toast of "The Association," said that if nurses were registered they would be armed with a sort of medical certificate, and trained medical men would have a fresh competition to meet. He thought that the evidence given before the Select Committee by representatives of that association would go a long way towards knocking on the head the proposal he referred to.

The President, in responding to the toast, said that there were many dangers threatening the medical practitioner. As for the registration of nurses, the state of things was bad enough already. In cases of illness people often sent for a nurse, and sent for a doctor only when the nurse had evidently failed, the patient by that time being often moribund. If this were so now, what would be done when nurses were registered? The association could not stop people forming societies and conferring titles on themselves, but it could do its level best to prevent Parliament from recognising them.

Dr. Hutchinson, M.P., in acknowledging the toast of "The Visitors," observed that the Select Committee on the registration of nurses would hold its last meeting for the reception of evidence next Tuesday. The public demanded that something should be done in this matter. The British Medical Association was said to have called for such registration by a unanimous vote; but they all knew what "unanimous votes" were. One witness representing the British Medical Association had urged before the committee that every nurse should undergo a State examination and should be punished for practising if not on the State register. That was an extreme view, but the public did want some protection, especially in regard to nurses from nursing institutions.

### Death in Our Ranks.

We regret to hear of the sudden death in Medellin, South America, of Miss Annie Goldsmith. For eight years Miss Goldsmith was district nurse at Swanage, where she was greatly esteemed.

### Westminster Hospital Ladies' Association.

The annual meeting of the Westminster Hospital Ladies' Association—postponed till later than usual this year because of the Historical Bazaar held in May—took place on Monday afternoon in the Board-room of the hospital. The Duchess of Buccleuch was in the chair, and after the minutes of the last meeting had been read, a most satisfactory announcement was made by Mr. J. Hautenville Cope, who has succeeded Miss Cave, lady superintendent of the hospital, as hon. secretary, she having asked to be relieved of the duties because of her time being fully occupied.

Mr. Cope stated that, owing to special efforts having been made by the members, 54 fresh names had been added to the association, including those of the Marchioness of Salisbury, Lady Esther Smith, the Duchess of Norfolk, Kathleen, Duchess of Westminster, Lady Edmund Talbot, and Mrs. Alfred Lyttleton, some of whom have consented to act as vice-presidents. The balance in the bank was £81, and it was agreed that £30 of this sum should be given to Miss Cave for the purchase of 15 new bedsteads for the patients in addition to the 25 already presented by the association. A further sum of £5 was voted to be handed over to Miss Cave for a necessitous case. A new development of the association is the appointment of eight ladies who have promised to undertake the care of the library books of the hospital, to visit the wards regularly, and with the assistance of the sisters, to give out new volumes to such patients as need them. They will also provide fresh literature, suitable for hospital use, when necessity arises, it being unanimously agreed that such books ought to be destroyed after three years' constant use.

The methods adopted by the ladies of the association to assist the hospital include subscriptions, gifts of flowers, fruit, clothes, and furniture, the organising of entertainments and fêtes to help the funds of the institution, the visiting of the wards and the aiding of the patients after they return to their own homes, and the sending of tickets for concerts, etc., to the nurses of the hospital.

### The Lord Chief Justice on District Nursing.

The annual meeting of the Kensington District Nursing Association took place on Tuesday in the grounds of Kensington Vicarage. H.R.H. Princess Louise, who was accompanied by the Duke of Argyll, attended the meeting.

The Duke of Argyll took the chair, and formally opened the proceedings.

The Vicar, Canon Pennefather, then proposed the adoption of the report for 1904, and the re-election of the Council, Lord Alverstone, Lady Phillimore, Lady Finlay, and Mrs. Percy Gates, Mayoress of Kensington, to be new members.

The report stated that the nurses of the Association paid £27,533 during 1904. The branch home established in Notting Dale had also been most successful. To show their appreciation of what had been done for them the poor people themselves voluntarily contributed over £50 during the last year towards the funds of the Association. Canon Pennefather referred in terms of the highest praise not only to the kindly administrations and valuable services of the nurses, but, quoting Mr. Charles Booth's words on district nursing, he observed that it could almost be said, "wherever a nurse enters the standard of life is raised." Last year, he added, the income of the Association was £21 below the expenditure, and he therefore very earnestly appealed for assistance, particularly increased annual subscriptions, for the continuance and increase of the Association work.

The Mayor of Kensington, Mr. Percy Gates, in seconding the motion, said that the Committee were very much to be congratulated not only upon the enormous number of cases attended by the nurses but also upon the fact that two-thirds of them had made complete recoveries. In the name of the poor he would like to thank the nurses. Referring to a letter of Miss Florence Nightingale's, written in 1896, he quoted her statement that she considered the work of a district nurse, if she kept a high standard, one of the great forces of civilisation. The Kensington nurses certainly kept a high standard. In addition they both rendered doctors indispensable assistance, and they taught the poor the meaning of fresh air, domestic economy, and the proper care of children. The municipal authorities looked upon the nurses as valuable co-workers.

The report having been unanimously adopted, the Lord Chief Justice moved: "That the Kensington District Nursing Association deserves hearty support," and expressed his own cordial support of the motion. Lord Alverstone, amidst laughter, said that he had a grievance against the vicar and the mayor for having between them appropriated all the best parts of his intended speech. No commendation, however, of the nurse's work was necessary, But in spite of their self-denial and devotion it could not be carried on without sufficient funds. If the wealthier classes would think of the contrast between illness in their own comfortable homes and in those of the struggling poor this charity could not fail to appeal strongly to them. We were all members of one great family, and it was the duty of those more highly placed to try and bring sunshine into the lives of their poorer brethren. The poor should be taught how to care for their sick children, and in these modern days of improved hygiene they should certainly be cautioned against all insanitary conditions. Over and over again at the assizes results of mothers' extraordinary ignorance in the feeding of their babies had come before him. This ignorance, which by no means came from callousness, the nurses' work to a very large extent counteracted. Not only were children's lives saved, but cleanly habits and other good influences inaugurated by the nurses continued to bear fruit long after the recovery of the patients. The poor could never really be taught from books, but only by intuition, and he could himself bear witness to the splendid influence of the nurses.

The motion was seconded by Dr. Rice-Oxley, who spoke of the immense value deservedly attached by medical men to the work of the district nurses—notably in the discovery and management of infectious cases whose existence were jealously guarded from the doctor's knowledge. In every circumstance the services of the nurse were all-important—indeed, the ultimate fate of the case depended upon her. He earnestly hoped that subscriptions would be forthcoming, for, although some might doubt the necessity for feeding the poor children, they were all of one mind when it came to the relief of their sufferings.

The motion was unanimously carried.

Cordial votes of thanks for the presence of the Princess Louise and the Duke of Argyll were then respectively proposed by Mr. Wyndham Gibbs and Canon Pennefather, and unanimously adopted. In returning thanks for the Princess and himself, the Duke of Argyll expressed his hope that those present would generously contribute to the collection to be taken at the conclusion of the meeting.

### Where to Go.

Friday June 30th: 11 a.m. till 1 p.m., 3 p.m. till 9.30 p.m.; 28 Victoria Street, S.W. Sale of articles not sold at the Westminster Hospital bazaar.

### Everybody's Opinion.

[Correspondence on all subjects is invited, but we cannot in any way be responsible for the opinions expressed by our correspondents. No communication can be entertained if the name and address of the correspondent are not given as a guarantee of good faith, but not necessarily for publication. All correspondents should write on one side of the paper only.]

### THE NURSE AND THE MASSEUSE.

"A Masseuse" writes: May I have a little space in which to air a real grievance, *i.e.* the average nurse apparently doing the work of the masseuse—some nurses are also qualified masseuses, and I do not allude to these? But is not massage a separate branch of work in the nursing world? I certainly understood it to be so when I paid a large fee to be trained in massage and the application of electricity, otherwise I would never have entered for it. Where does the fault lie? Does the average nurse profess to understand massage because she has had to do rubbing while training as a nurse in hospital? or would a medical man consider such rubbing sufficient grounds for sanctioning her to apply such treatment? If so, what is to become of the masseuse proper? I often hear it said, "Oh, the nurses do the rubbing now." I wish to give a few reasons why this unjust practice should not be continued. Ordinary rubbing cannot possibly do as much good as the skilled movements, therefore such rubbing, if harmless to the patient, is detrimental to such treatment as massage. Why should the trained nurse, who, as a rule, has enough to do, take the duties of a masseuse, whose work at all times is most uncertain? Is any nurse while on duty fresh and strong enough to rub at all with any benefit?

### THE NEED FOR THE REGISTRATION OF PRIVATE HOMES.

"Fair Play" writes: I have read with interest "A Victim's" letter on the need for registration of nursing homes, and I can only say that her experience of the nursing home she alludes to has been quite the reverse of what all other nurses I know have generally experienced. For myself, I was over four years in one Home and about a year and a half in another, both run on the co-operation principle, and I have been very satisfied with my treatment and the fees, etc., charged to me. It has been my experience that the more the Home does for the nurses the less they appreciate it. I think that the writer of the letter in question fully shows this. She says that when she is living on full board, lodging, and attendance at the Home she only pays 14s. per week, which I presume includes bedroom with clean bed-linen, four meals per day and also the use of a good sitting-room, with no doubt use of a piano and telephone. Where does she expect to get such accommodation elsewhere for the same money? Surely the 4s. per week charged when she is at cases she does not grudge towards the upkeep of the premises. The matron of the Home must expect to get something towards rent, rates, taxes, wear and tear of furniture, washing of table and bed-linen, etc., or perhaps "A Victim" thinks that the Home should be run entirely for her benefit and be at her free disposal whenever she wishes to use it, never mind who pays for it. As regards the earning of only £15 15s. during the 12 months she was at the institution, I think that in this item she practically condemns herself. How could she expect to take on cases for the Home when she owns that she was already engaged on her own cases? No doubt, if the truth were known, she could have had cases if she had not taken her own on. I should also like to know how many cases she refused to attend from the Home. There is such a thing as nurses picking and choosing their cases and then blaming the Home for not providing work, and probably "A Victim" is well aware of this. It seems very strange to me that "A Victim" stayed so long in a Home where she was so badly treated, and whose terms she should have known before going there, and owns if she left there she would most probably have gone to another, when she is so anxious that some legislation should make it impossible for such Homes to exist. I cannot see what sort of inspection she would like to see established, nor why a system that satisfies all but a few malcontents should be hampered in any way. To abolish these Homes would probably throw a great many deserving nurses out of work, for "A Victim" candidly admits that it is equally difficult sometimes for a nurse to obtain work on her own account.

### NURSES AND MONEY-MAKING INSTITUTIONS.

"Essex" writes: There is doubtless a good deal of truth in what an "Institution Matron" says of nursing institutions. But there is also a great deal to be said on the other side. I, for one, could tell of the far from right methods adopted by nursing home matrons towards their nurses. So far as my experience went nurses were only given consideration as they earned money for the matron, and while doing this they were often at the same time paying for their board and lodging. I took on trust an offer to be trained in district work with a promise of employment at the end of 13 weeks' engagement, for which I paid 13 guineas. I found within a few days that I—with other nurses who had come on the same terms—was expected to earn money for the home. I was sent on a private mental case for which two guineas a week was charged, the patient finally coming into the home. Other cases of a medical character followed and I scarcely touched district work. However, I got on well and gave satisfaction to both doctors and patients. After five months' service at the home I took the parish work of a little town in Berkshire where a nurse was only employed for the winter months. The matron then asked me to come back on her staff. Liking the neighbourhood and being almost continually out on cases I stayed on for a time, till I could stand it no longer. The discomfort of the average nursing home and the utter want of consideration for the nurse are most trying. Another bad practice, which I fear is only too common, is that of making nurses leave cases and go on to others, a practice both upsetting to the patient and vexatious to the nurse. On several occasions I had, in the interest of the patient, to refuse to obey the matron's orders to leave one case unfinished and go on to another, and I find I am not alone in my experience. Certainly a nursing home can be conducted on business lines, and should be made to pay, in fact, nothing pays better, but the sweating system now so common ought to be thoroughly exposed. "Sympathiser" is right in her assertions. Where a nursing home is established on businesslike, charitable, and honest lines no greater blessing can come into a neighbourhood. There are some so conducted. Doubtless "An Institution Matron" is associated with one of these. I wish there were more of them.

### Appointments.

Corbett General Hospital, Stourbridge.—Miss Isabel Gill has been appointed staff nurse. She was trained at the Royal County Hospital, Guildford.

Derby Workhouse Infirmary.—Miss Alice Hewson has been appointed staff nurse. She was trained at Hunslet Union Infirmary.

Leicester Workhouse Infirmary.—Miss A. B. Clarke has been appointed matron. She was trained at the City Hospital, Birmingham, and Birmingham Infirmary. She has since been home sister, night superintendent, and assistant matron at Stockport Infirmary, superintendent and matron at Shoreham Infirmary, and matron at Portsmouth Infirmary.

Mitford and Launditch Workhouse Infirmary.—Miss M. Plumb has been appointed superintendent nurse. She was trained at West Ham Infirmary, where she was afterwards staff nurse. She has since been staff nurse at Derby Union Infirmary.

North Staffordshire Infirmary, Stoke-upon-Trent.—Miss Jane McMaster has been appointed superintendent nurse. She was trained at St. Thomas's Hospital, London, where she was successively staff nurse, assistant night superintendent, and sister. She has since been matron of Salisbury Infirmary.

Royal Berkshire Hospital, Reading. — Miss Margaret McDougall has been appointed sister. She was trained at the Royal Infirmary, Edinburgh, and has since been sister at the Samaritan Hospital for Women, Glasgow.

Salisbury Infirmary. — Miss E. M. Vezey has been appointed matron. She was trained at the Nightingale School, St. Thomas's Hospital, London, where she was afterwards sister. She has since been matron of Savernake Hospital, Wiltshire, and assistant matron at St. Thomas's Hospital.

Samaritan Free Hospital for Women, London.—Miss Florence Tice has been appointed matron. She was trained at St. Bartholomew's Hospital, London. She has since been sister at the Birkenhead and Wirral Children's Hospital, and night superintendent and sister at University College Hospital, London.

Shepton Mallet Hospital and Dispensary.—Miss Ida Mackintosh has been appointed matron. She was trained at Sunderland Infirmary, and has since been district nurse for the North London Nursing Association, matron of Ascot Cottage Hospital, Elder Hospital, Govan, Glasgow, and Queen's Jubilee Hospital, London.

Telfair Hospital, Savannah, United States of America.—Miss E. M. Banner has been appointed matron. She was trained at the Norwich Isolation Hospital, the Brook Hospital, Shooter's Hill, and the Hull Royal Infirmary, where she has since been sister of the men's medical and surgical wards, and subsequently night superintendent.

Thetford Cottage Hospital.—Miss Elizabeth Johnson has been appointed matron. She was trained at Norfolk and Norwich Hospital, where she has since been theatre nurse and temporary sister. She has also been sister in charge of the female ward at the Royal Buckinghamshire Hospital, Aylesbury.

Tredegar Park Cottage Hospital.—Miss Frances M. Hollister has been appointed matron. She was trained at Cardiff Infirmary.

Victoria Hospital, Folkestone.—Miss Eva Pedley has been appointed matron. She was trained at the Royal Free Hospital, Gray's Inn Road, London. She has since been head nurse at the Devonshire Hospital, Buxton; night sister, ward sister, and theatre sister at the District Hospital, Grimsby; sister of the male ward, theatre sister, and temporary matron at the Victoria Hospital, Folkestone.

Woolwich and Plumstead Cottage Hospital.—Miss E. England has been appointed staff nurse. She was trained at Salford Royal Hospital, and has since been nurse at Ripon Cottage Hospital, and sister at Salford Royal Hospital.

### Novelties for Nurses.

(By Our Shopping Correspondent.)

### ICILIMA SOAP AND FLUOR CREAM.

These two specialities of the Icilima Company, 142 Gray's Inn Road, will be found most acceptable for summer use. The basis of both is the water from the famous Selama artesian spring in Algeria, which possesses special properties beneficial to the skin. The soap is a very pleasant and well-made Castile soap, and the Fluor cream well deserves the description given of it by the makers. It has an unusually refreshing effect, and leaves no oily trace behind it. It is declared free from zinc or bismuth.

### NURSING REQUISITES AND SURGICAL APPLIANCES.

The Hospitals and General Contracts Company, of 33 Mortimer Street, have issued a very complete illustrated catalogue dealing with the above. This company is making a special study of nurses' requirements, and a perusal of the catalogue demonstrates the importance they attach to the subject. The instruments are those which the nurse has either the charge of or uses in the course of her work. Bandages, dressings, plasters, chloroform drop-bottles, ether apparatus, district nurses' bags, midwifery bags, pincushions, wallets, invalid furniture, portable baths with apparatus for the different methods of application, air-beds, pillows, invalid furniture and other items, form the wide scope of the catalogue. The articles supplied are of good quality and are not expensive, and the list is one which can be usefully retained for reference.

### NORWEGIAN SARDINES.

The sardines I wish to introduce to our readers are called "Skipper" sardines. Not much attracted by the grand name, I performed my duty of tasting them with some misgiving. The doubt was wholly unnecessary, and henceforward if I can obtain "Skipper" sardines I shall nct purchase any others, for in my estimation they are superior to any others of which I have had experience. These sardines are caught only in the Norwegian Stavanger fiords, we are told, and are preserved in the purest olive oil by the purveyors, Chr. Bjelland and Co. In flavour and substance they are different from, and much more delicate than, the usual sardines sold. There is no strong taste of oil, and the only fault I can find with them is that they are so small and so tempting as to make a moderate consumption of them difficult. They are sold or can be procured by all grocers.

### TRAVEL NOTES AND QUERIES.

By our Travel Correspondent.

Convent near Lille (Oakdene).—Many thanks for useful information. I will write.

Cheap Quarters at Harrogate (Alma).—I fear that you will find nothing at Harrogate or Buxton at your terms, but write to the managers at both London termini and ask them to send you their gazettes with lists of boarding-houses and lodgings.

Minehead at 25s. per Week (Maria).—I fear that such ideal accommodation is not to be had, but write to the manager at the London terminus and ask for the Gazette with the list of lodgings, etc., on the line. There is a quiet place called Burnham that might suit you. It is on the sea, but not pretty like Minehead. Yes, there are coach drives from Minehead.

Venice via Berne (Beggar).—Yes. Dr. Lunn, 5 Endsleigh Gardens, W.C., arranges parties on very cheap lines, but of course you must adhere to dates and route. The usual way and the cheapest is by Bale, Milan and Verona. From London second class return about £8. If you wish to join the party from Berne why not join it at Lucerne, it is not more than 40 miles from Berne.

Cycling Tour in Belgium (J. M. B.)—You cannot do a tour for a fortnight for £5, because change of hotels is very expensive. You can manage a fortnight only by going to one place and stopping there, seeing all the surrounding country. This would be interesting either at Bruges or Knokke. A longer journey cannot be arranged. If you like this idea let me hear again which you prefer as a centre and I will advise you further.

Fontainebleau for a Week (E. A. A.).—The amount is very small but I think it may be done if you travel third class. Return to Paris *via* Newhaven and Dieppe by night service, third class, £1 13s. 3d. Return fare on to Fontainebleau about 7s. Unfortunately Fontainebleau is a very expensive place. Try Hôtel du Nord et de la Poste and ask if they will take you on the third or fourth floor for a week at 7 frs. per day, this will be another £2, leaving only £1 for tips, omnibuses, etc. It is rather close work, but perhaps you can squeeze out another £1. Take only a large handbag. It will be enough for a week and save you cabs and porters.

### Notes and Queries.

**REGULATIONS.**

**The Editor is always willing to answer in this column, without any fee, all reasonable questions, as soon as possible.**

**But the following rules must be carefully observed.**

1. **Every communication must be accompanied by the name and address of the writer.**
2. **The question must always bear upon nursing, directly or indirectly.**

**If an answer is required by letter a fee of half-a-crown must be enclosed with the note containing the inquiry.**

*Rhinitis.*

(104) Will you kindly tell me the danger likely to arise from a too excessive use of adrenalin as a nasal spray? The case is one of rhinitis.—*Pros and Cons.*

The use of adrenalin can only be safely practised under medical supervision.

*India.*

(105) I want to know if Lady Dufferin's nurses and Lady Roberts' nurses are one and the same, or quite separate, also have they anything to do with the Up-Country Nursing Association for India. Can you tell me to whom to apply for each?—*Hants.*

All three associations are quite separate. Lady Dufferin's Fund does not employ nurses, and Lady Roberts selects her nurses from amongst the Army Sisters. For the Up-Country Nursing Association apply to Hon. Secretary, Dalkeith House, Cambridge Park, Twickenham.

*Ceylon.*

(106) Will you kindly give me the address of a private nursing home in Colombo, Ceylon?—*Pattie.*

We know of no private nursing home in Ceylon. There is a Ceylon Nurses' Association in Colombo which supplies private nurses.

*France.*

(107) Will you oblige me with the addresses of some good nursing homes in France—Paris preferred?—*E. M. M.*

You have omitted to give your address, but we have no record of private nursing homes in France.

*Imbecile.*

(108) Can you please tell me of a home for a poor imbecile child? Mother died recently. Father would pay small sum weekly.—*A. J. C.*

Write to the Secretary of the National Training Home for the Feeble-minded, 36 King William Street, London, E.C.

*Serum Treatment.*

(109) Will you kindly inform me where I can get information on the serum treatment for phthisis, or is there a pamphlet printed on that subject?—*L. G.*

A chapter on antitubercular serums will be found in "Serums, Vaccines, and Toxines" by W. Cecil Bosanquet, M.D., published by Cassell and Co. It can be obtained from the Scientific Press.

*Convalescent Home.*

(110) Will you kindly publish the addresses (mentioning fee, if possible) of any convalescent home on the East Coast where a tired nurse could get rest and change at a low fee?—*Violet.*

Write to the Lady Superintendent, Claughton Home, Walton-on-Naze, Essex, for full particulars.

*India.*

(111) I should be glad if you would kindly tell me the best place to apply for a post as lady nurse to some lady going out to India, or some such place, who would require some one to take charge of her baby or children on the voyage out, and to stay awhile out there?—*H. A. T.*

You had better advertise.

*Midwifery Training.*

(112) Will you kindly tell me if there is a hospital where I can obtain a free midwifery training?—*C. H.*

Write to the Secretary, the Rural Midwives Association, 47 Victoria Street, Westminster, S.W., and inquire if help can be given.

**Handbooks for Nurses.**

| | Post Free. | |
|---|---|---|
| "How to Become a Nurse: How and Where to Train." | 2s. | 4d. |
| "Nursing: its Theory and Practice." (Lewis.) ... | 3s. | 6d. |
| "Nurses' Pronouncing Dictionary of Medical Terms." ... | 2s. | 0d. |
| "Complete Handbook of Midwifery." (Watson.) ... | 6s. | 4d. |
| "Preparation for Operation in Private Houses." ... | 0s. | 6d. |

**Of all booksellers or of the Scientific Press, Limited, 28 & 29 Southampton Street, Strand, London, W.C.**

### For Reading to the Sick.

"SORROWFUL YET REJOICING."

Trust thou ever in Him nor fear,
By day nor by night;
In the storm will His presence appear,
In darkness His light.
He shall come to thee fair as the dawn,
In gladness of grace;
Thou shalt see, at eve and at morn,
The light of His face.

He bringeth the breath of His love,
As peace after pain,—
As the fragrance of meadows above
The tumult of rain.
He giveth His Joy for the day,
His Rest for the night;
He leadeth His loved by the way,
Till Faith becomes Sight!

*Kingsland.*

"Rejoice in the Lord alway: and again I say, rejoice." Think only when and where those words were written, in apparent failure, from the prison at Rome! We read of the Apostles, how, beaten, threatened, and misunderstood, "they departed from the presence of the council, rejoicing that they were counted worthy to suffer shame for His Name." Yes, Joy is a very needful gift, a very blessed gift, little valued, little sought for, but still one of those gifts of the Spirit which constitute the very refinement of Christianity.

There is a Joy, the peculiar property of the soul, which hangs with a pervading fragrance round the writings of the saints and their books of devotion, so much so, that sometimes their words seem strange and unreal to our tender hearts; a Joy which indicates a satisfaction which the world can neither give nor take away.

We might further describe Joy as the radiant atmosphere which plays around pleasure: and if pleasure is, roughly speaking, satisfaction, and the highest pleasure the highest satisfaction, Joy will be the illumination, half conscious, half unconscious, which plays about the life of true pleasure.

Joy has its distinguishing marks and characteristics as well as "Love," the freshness and verdure which mark out its course. And one of these surely will be hopefulness; joyful through hope. How many a man has surmounted apparently insuperable obstacles, because Joy has whispered to Hope and Hope has said, "It can be done."

These surely are but expressions of the same great thought, that the fruit of the Spirit is Joy. And where there is only gloom and sadness instead of brightness and light, there must then be some other agency at work, which loveth darkness rather than light, not the joyous agency of that Holy Influence, Whose "blessed unction from above, is comfort, life, and fire of Love."—*Canon Newbolt.*

O Lord, happy is the time
When in Thy love I rest;
When from my weariness I climb
Even to Thy sacred breast.
The night of sorrow endeth there;
Thou art brighter than the Sun,
And in Thy pardon and Thy care
The heaven of heavens is won.

*Translated by G. Macdonald.*

